# The Use of N-Terminal-Pro-BNP in Preterm Infants

**DOI:** 10.1155/2009/175216

**Published:** 2009-12-30

**Authors:** Afif EL-Khuffash, Eleanor Molloy

**Affiliations:** ^1^Department of Neonatology, Hospital for Sick Children, Toronto, ON, Canada M5G 1X8; ^2^Department of Postgraduate Research, School of Medicine and Medical Sciences, University College Dublin, Dublin 4, Ireland; ^3^Department of Neonatology, National Maternity Hospital, Dublin 2, Ireland; ^4^Department of Neonatology, Lady's Children's Hospital, Crumlin, Dublin 12, Ireland

## Abstract

The use of natriuretic peptides in the neonatal population is emerging. B-type Natriuretic Peptide (BNP) and N-terminal-Pro-BNP (NTpBNP) are used in the adult population to assess myocardial function and volume loading. Their role in prognosis following cardiac surgery has also been identified. In preterm infants NTpBNP is becoming increasingly recognised as a potential screening tool for patent ductus arteriosus (PDA), and a marker for myocardial performance. In addition, NTpBNP may provide prognostic information in preterm infants and term infants with congenital diaphragmatic hernia (CDH). In this paper, the role of NTpBNP in the preterm population will be discussed.

## 1. Introduction

B-type Natriuretic Peptide (BNP) is a 32-amino acid ring structure coded by a sequence on chromosome 1. BNP acts on a cyclic guanosine monophosphate (cGMP) coupled receptor via a transmembrane domain [1]. The ventricles of the heart are the main site of BNP synthesis and release in response to volume loading, pressure loading, and ventricular stress. BNP causes diuresis, natriuresis, arterial and venous vasodilatation, and antagonises the renin-angiotensin system. The net effect is a reduction of intravascular volume, ventricular preload and afterload. The ring structure must remain intact to ensure receptor binding to natriuretic peptide receptors A and B (NPR-A/B). BNP is excreted following cleavage by membrane bound neutral peptidase found in the kidneys and vascular tree. NPR-C also clears it from the blood by binding, endocytosis, and lysosomal degradation [2]. N-terminal-Pro-BNP (NTpBNP) is the inactive by-product of BNP resulting from the cleavage of the parent peptide Pro-BNP. Both are released into the circulation but no function has been associated with NTpBNP. BNP has a short half-life of 20 minutes and is unstable at room temperature. NTpBNP has a longer half-life of compared to 60 to 120 and is stable under a range of storage conditions. There are multiple kits available for BNP measurements. This has led to discrepancies in cut off levels across studies used for assessing BNPs prognostic and haemodynamic assign properties. On the other hand, available kits used for NTpBNP measurement are limited in number. This may allow more comparisons to be made across studies, thereby rendering it more suitable than BNP for routine clinical monitoring [3]. 

## 2. Clinical Role of Natriuretic Peptides

BNP and NTpBNP are good screening tools, detecting chronic ventricular dysfunction in adults, with sensitivities and specificities surpassing clinical and radiological methods [4]. BNP and NTpBNP are useful in the diagnosis of congestive heart failure in dyspnoeic patients presenting to the emergency department [5]. In addition, these markers facilitate the screening, treatment response, and prognosis of asymptomatic patients with subclinical Left Ventricular dysfunction [6–8]. 

BNP and NTpBNP levels are elevated in children with heart disease causing ventricular pressure and volume loading [9, 10]. In addition, plasma BNP correlates closely to shunt volume in left-to-right cardiac lesions, increasing with decreasing left ventricular ejection fraction, and positively correlating with increasing right ventricular systolic pressures [11]. The levels can also reflect functional capacity in children with congestive heart failure [9]. In children with dilated cardiomyopathy NTpBNP is a good marker for persistent left ventricular dysfunction with levels normalising in children whose echocardiograms recover [12]. Among infants with respiratory distress, plasma NTpBNP measurement can differentiate between acute heart failure and lung disease and can be used to monitor response to treatment [13]. Elevated preoperative NTpBNP in children undergoing open heart surgery is linked with complicated postoperative outcome [14]. NTpBNP is used preoperatively to predict the development of postoperative low cardiac output syndrome (LCOS) [15]. BNP levels tend to be higher in children receiving long-term immunosuppressive treatment post liver transplant compared to healthy controls despite the lack of echocardiographic evidence of cardiac compromise suggesting that BNP levels may identify patients with early cardiac damage [16].

Limited data have demonstrated the potential benefit of these peptides as markers of cyanotic and obstructive lesions. Cowley et al. demonstrated a significant, correlation between left ventricle to aorta gradient and BNP levels [17]. NTpBNP has also been used to monitor response to valve replacement in patients with aortic stenosis [18]. In a recent study, Hopkins et al. found higher levels of NTpBNP in 10 adult patients with cyanotic heart disease (including Eisenmenger's syndrome) despite the lack of ventricular pressure loading [19]. 

## 3. Application of NTpBNP in Neonates

BNP and NTpBNP levels surge at birth to reach a plateau on days 3 to 4, followed by a steady fall to reach a constant level in infancy [20]. This surge is probably multifactorial but may be due to the loss of the placental low pressure system and exposure to the initially suprasystemic pulmonary pressures subjecting the ventricle to greater volume and pressure loading. Furthermore, the placenta plays a role in clearance of NPs and loss of this clearance system contributes to the high level [21]. This surge in BNP levels at birth may play a regulatory role in the haemodynamic changes associated with transition to extra-uterine life. Renal maturation, a rise in systemic vascular resistance and a fall of pulmonary pressures explain the subsequent fall in peptide levels. 

There is a paucity of normative values of NTpBNP in neonates (Table 1). Reference ranges quoted in the literature vary according to the timing of the test, the kits used, and the population investigated [22]. Most quoted reference ranges are for term healthy neonates and therefore do not represent the intensive care population. NTpBNP is not thought to cross the placenta and therefore any variation in neonates must be explained intrinsically [23].

## 4. Influence of Antenatal and Postnatal Events on NTpBNP Levels in Preterm Infants

The use of NTpBNP in assessing the haemodynamic status of preterm infants is gaining interest. NTpBNP is released in equimolar amounts to BNP from the myocardium. Its levels however are higher and remain in the blood stream for longer due to differing half-life and clearance [24]. Levels of NTpBNP in the early preterm period were assessed in a study of 80 preterm infants with a median gestation of 28 [IQR 26.1–29.5] weeks and median birth weight of 1.06 [IQR 0.87–1.21] kg. At 12 hours of life all infants had a PDA with low velocity left to right shunting. The median NTpBNP value for the cohort was 1273 pmol/L with an interquartile range (IQR) of 664–2798 pmol/L and a range of 98–10 700 pmol/L. The influence of antenatal and postnatal factors on NTpBNP levels is illustrated in Table 2. Infants with RDS had significantly higher NTpBNP values compared to controls [25]. When adjusted for RDS, gestation and birth weight had no impact on NTpBNP. The premature neonatal heart is distinguished from that of older infants by several unique characteristics. The neonatal myocardium has a higher water concentration and a greater proportion of “stiff” collagen resulting in a noncompliant ventricle and diastolic dysfunction resulting in relatively poor ventricular filling [26]. The preterm myocardium cannot therefore respond to stress caused by the rise in afterload following the loss of the low pressure system of the placenta. This problem is further compounded by any potential stressors such as hypoxia, anaemia and, mechanical ventilation which reduces venous return and cause pressure on the myocardium preventing effective contraction [27]. This may explain the higher values of NTpBNP seen in preterm infants compared to term infants. 

At 12 hours of life, there is a significant negative correlation between NTpBNP and echocardiographic markers of left ventricular function including mean velocity of circumferential fibre shortening (mVcfs) (*r* = −0.32, *P* = .02), and shortening fraction (*r* = −0.26, *P* = .03). Interestingly, there is no correlation between NTpBNP and left atrial to aortic root ratio (LA : Ao) or the diameter the PDA at 12 hours of life (*r* = −0.14, *P* = .24). Echocardiographic assessment of the effect of ductal shunting on the systemic and pulmonary circulations suggests that shunting across the PDA is not significant at this early stage due to the relatively high pulmonary vascular resistance [28]. 

NTpBNP levels were assessed in the same population of infants at 48 hours of age [29]. Forty five infants developed a PDA compared to 35 infants who closed their ducts spontaneously beyond 48 hours. Infants with a PDA had a significantly higher NTpBNP levels compared to controls (6059 versus 740 pmol/L, *P* < .001). There is a significant strong correlation between NTpBNP and LA : Ao (*r* = 0.49, *P* ≤ .001), PDA diameter (*r* = 0.54, *P* = .001). NTpBNP provided information regarding the effects of the PDA on pulmonary overcirculation, represented by a positive correlation between levels and a rising left ventricular output (LVO) (*r* = 0.31, *P* = .006) and an increasing mitral valve inflow signal (*r* = 0.34, *P* = .007) assessed by echocardiography. In addition, NTpBNP provides information regarding systemic hypoperfusion. This was represented by a negative correlation with descending aortic end diastolic velocity (*r* = −0.58, *P* = .001), and celiac artery blood flow (*r* = −0.41, *P* = .001). A Receiver operating characteristics curve (ROC) was constructed to evaluate the bioassays' detection of a PDA confirmed by echocardiography, with an area under the curve (AUC) of 0.88 (95%CI 0.79–0.96) for NTpBNP. At 4000 pmol/L, NTpBNP had a sensitivity of 70% and a specificity of 89% for the presence of a PDA [35]. 

The association between NTpBNP and PDA levels was replicated in three other studies (Table 3). The cut-off levels for the detection of a PDA vary by 2- to 3-fold amongst the studies (Table 3). This may be due to the different populations studied and the differing definition of a haemodynamically significant PDA across the studies. In the three other studies, no echocardiographic assessments were carried out to assess systemic hypoperfusion and pulmonary circulation [36–38]. They relied solely on ductal diameter and LA : Ao ratio. These markers in isolation do not give an accurate assessment of the degree of ductal steal and pulmonary over circulations. Therefore, the use of a lower threshold may over diagnose a significant PDA [28, 29]. Nuntnarumit et al. found a stronger correlation with LA : Ao (*r* = 0.77, *P* = .001). The lack of effect of gestation and birth weight on NTpBNP levels were also demonstrated [36]. Interestingly, Farombi-Oghuvbu et al. found a weak negative correlation between gestational age and NTpBNP levels on day 1 (*r* ^2^ = 0.16, *P* = .02). The lack of influence of antenatal and postnatal factors illustrated in Table 2 was replicated in their study [37]. They showed a lack of association between NTpBNP levels and prolonged rupture of membranes, the use of inotropes, and early sepsis. They did find however that there was a weak association between gestational age and NTpBNP with the levels being higher in more premature infants (*β* = −0.495, *P* = .013). 

NTpBNP may have a role in monitoring treatment response. In our population, following successful PDA treatment, NTpBNP levels fell significantly to levels similar to controls (from 6059 to 998 pmol/L, *P* < .001). In the control group NTpBNP levels decreased significantly from 740 pmol/L at 48 hours to 272 pmol/L on Day 7. Nuntnarumit et al. showed that infants who do not respond to the initial course of treatment have persistently high NTpBNP levels compared to responders (2337 versus 353 pmol/L, *P* = .007). 

The ability of NTpBNP to predict the presence of a PDA seems to surpass that of BNP. There is a wide variation in BNP levels associated with a significant PDA ranging from 70–1110 pg/mL despite using the same assay [39–42]. This may be explained by the shorter half-life and instability of BNP described earlier. In addition, the correlation of NTpBNP with the echocardiographic markers of PDA significance is more consistent. Flynn et al. showed that BNP had a weaker correlation with echocardiographic markers of PDA significance including ductal size (*r* = 0.62), increased pulmonary flow (*r* = 0.63), and increased steal (retrograde diastolic flow) in the descending aorta (DAo) and the superior mesenteric artery (SMA) (*r* = 0.54 and 0.41). There was a poor correlation between left atrial to aortic ratio (LA : Ao ratio) and BNP levels (*r* = 0.33) in this study [43]. However, Choi BM et al. revealed a stronger correlation (*r* = 0.73) [39]. 

NTpBNP may be an ideal screening tool for a PDA due to relatively close cut-off levels of NTpBNP for diagnosis a PDA across the studies conducted thus far, the strong correlation with echo markers of PDA significance, and the lack of influence of antenatal and postnatal factors on the levels. In addition, the levels fall significantly following successful treatment and persist in nonresponders. This obviates the need for repeated echocardiograms to assess treatment success. 

## 5. NTpBNP and Outcome

The ability of NTpBNP to predict short-term outcomes in preterm infants with a PDA was assessed by El-Khuffash et al. [29]. In the study described in the previous section, the PDA group was subdivided into infants with a poor outcome (grade III/IV IVH, death or both, *n* = 20) and infants without complications (*n* = 25). The poor outcome group had a significantly lower gestation and birth weights. There were no differences in the antenatal characteristics between the two groups. Infants in the poor outcome group had a significantly higher NTpBNP levels at 48 hours compared to infants without PDA-associated complications (9284 pmol/L [5013–16 911] versus 5121 pmol/L [2324–6202], *P* = .008). The AUC for NTpBNP's ability to predict severe IVH and/or death as a complication of a PDA is 0.84 (95% CI 0.72 – 0.96, *P* ≤ .001). A level of 5500 pmol/L has a sensitivity of 80% and a specificity of 80%. Only one infant in the Spontaneous PDA closure group died before discharge. The 12- and 48-hour NTpBNP levels were 2023 pmol/L and 6605 pmol/L respectively. NTpBNP may be an independent marker of poor neonatal short-term outcome irrespective of PDA presence (29).

Gagliardi L et al. assessed the discriminatory ability of the clinical risk index for babies (CRIBs), CRIB-II, and SNAPPE-II in detecting death before discharge in 720 preterm infants [44]. Following the exclusion of babies weighing 400–499 g (*n* = 15), the AUCs for CRIB, CRIB-II, and SNAPPE-II were 0.898, 0.905, and 0.835, respectively. These results were comparable to the AUCs for NTpBNP and death in this cohort. NTpBNP may prove to be a useful adjunct to clinical and echocardiographic PDA staging system proposed by McNamara et al. [45]. Medical therapy for PDA has well recognised adverse effects and neither prophylaxis nor treatment on the basis of clinical and echocardiographic signs have been shown to improve long-term outcomes. Accurately identifying infants with PDA who are at highest risk of poor outcome using NTpBNP may allow more successful trials of targeted medical therapy of PDA.

## 6. Other Applications of NTpBNP

Pulmonary vascular resistance may remain elevated during the neonatal period leading to difficulties in oxygenation and resulting in pulmonary hypertension (PHT). Echocardiography is required to distinguish PHT from other respiratory and cardiac disorders by demonstrating suprasystemic pulmonary vascular pressures. In a study of 28 term infants, Baptista et al. showed a significantly higher NTpBNP level in infants with PHT secondary to congenital diaphragmatic hernia (CHD) compared to age and weight matched controls (1563 versus 591 pmol/L, *P* < .05). There was a good correlation with right ventricular mean pressure (*r* = 0.45, *P* = .03) and RV Tei index. This measurement is a combined myocardial performance index (isovolumic contraction time plus isovolumic relaxation time divided by ejection time) (*r* = −0.46, *P* = .02). In addition, the prognostic properties of NTpBNP are demonstrated in this trial. Nine infants in the CHD group died before discharge. NTpBNP was higher in the nonsurvivors (2679 versus 737 pmol/L, *P* = .009). A level of 1360 pmol/L had a 100% sensitivity and 67% specificity for identifying these infants. 

Plasma BNP increases in animal models with induced endotoxaemia and the proinflammatory cytokine inter leukin-6 (IL-6) has been linked with BNP production. Therefore, the rise of BNP may not be solely due to ventricular overloading. In neonatal rat cardiac myocytes, transcriptional activation of the BNP gene was initiated by lipopolysaccharide (LPS) suggesting that elevated BNP levels under endotoxaemic conditions are partially mediated by LPS [46]. In patients with severe sepsis or septic shock, BNP and NTpBNP values are highly elevated [47, 48] and, despite significant hemodynamic differences, comparable with those found in acute heart failure in adult patients. It remains to be determined how elevations of natriuretic peptide levels are linked to inflammation and sepsis-associated myocardial dysfunction [37, 48]. NTpBNP may also serve as useful laboratory marker to predict survival in patients presenting with severe sepsis [49]. Additionally, NTpBNP seems to be an early predictor of myocardial dysfunction in patients with septic shock [50]. NTpBNP may serve as a marker of cardiac dysfunction associated with sepsis in preterm neonates and be a useful adjunct in the diagnosis of sepsis. In preterm infants, NTpBNP rises in the presence of late onset sepsis without the presence of a PDA or ventricular dysfunction. The interpretation of NTpBNP levels in the presence of a PDA and sepsis warrants further study.

## 7. Conclusion

NTpBNP has a major diagnostic role in the adult population. In children, NTpBNP serves as an indicator of cardiac disease and may be used to monitor response to treatment. The potential benefit of these NTpBNP in neonatology is immense. It has a role in PDA screening, treatment response and may also offer prognostic information. More studies are needed to explore the possible roles of NTpBNP in the management of sepsis and monitoring of cardiac performance. These two possible confounding factors may limit its reliability in the diagnosis of PDA and its response to treatment.

## Figures and Tables

**Table 1:** Reference ranges for NTpBNP.

Study	N	Age range/source	Kit	NTpBNP levels
Mir et al. [20]	153	Day 1	Biomedica	Mean: 641 pmol/L
		Venous/cord		Range: 254–1272
Mir et al. [20]	153	Day 3	Biomedica	Mean: 246
		Venous/cord		Range: 110–430 pmol/L
Nir et al. [22]	43	0–2 days	Elecsys	Median: 376 pmol/L
			1010/2010	Range: 30–1560 pmol/L
Bar-Oz et al. [23]	122	Day 1	Elecsys	Mean: 68 pmol/L
		Cord blood	1010/2010	SD: 41 pmol/L
Bar-Oz et al. [23]	33	Day 1	Elecsys	Mean: 359 pmol/L
		Plasma	1010/2010	SD: 210 pmol/L
Schwachtgen et al. [30]	62	Day 1	ECLIA	Mean: 97 pmol/L
		Cord blood	Roche	Range: 33–306 pmol/L
Hammerer-Lercher et al. [31]	42	Day 1	Elecsys	Mean: 65 pmol/L
		Cord blood	1010	IQR: 49–98 pmol/L
Bakker et al. [32]	67	32–42wk	Elecsys	Mean: 79.5 pmol/L
		Cord blood	2010	SD: 42.9 pmol/L
Rauh and Koch [33]	13	<1 month	Elecsys	Range: 132–913 pmol/L
		Plasma		
Soldin et al. [34]	40 *♂*	<31Days	Dade RxL	97.5th percentile: 3352 pmol/L
		Plasma	Dimension	
Soldin et al. [34]	53 *♀*	<31days	Dade RxL	97.5th percentile: 4940 pmol/L
		Plasma	Dimension	
EL-Khuffash et al. [25]	80	12 hours of life	Elecsys	Mean: 1273 pmol/L
		<1500 grams	2010	IQR: 664–2798 range: 94–10700

**Table 2:** Influence of antenatal and factors on NTpBNP levels at 12 hours (Mann Whitney U test was used to compare medians).

Grouping variable	Group	NTpBNP (pmol/L)	
		Median	*P*-value
Sex	Boy	1273	.32
	Girl	1454	
Chorioamnionitis	No	1199	.07
	Yes	2248	
Steroids	None	1586	.42
	Partial	840	
	Full	1462	
Delivery	SVD	1275	.60
	CS	1222	
Inotrope use	No	1368	.83
	Yes	1269	
RDS	No	815	.04
	Yes	1391	

**Table 3:** The use of NTpBNP in PDA diagnosis. NTpBNP rises significantly in the presence of a PDA. All studies used the Roche Elecsys system. ROC: Receiver Operating Characteristics Curve; *N* no PDA: number without PDA; *N* PDA: Number with PDA.

Study	Gestation mean (wks)	Birth weight mean (Kg)	Day of life	*N* no PDA	NTpBNP (pmol/L)	*N* PDA	NTpBNP (pmol/L)	Cut off NTpBNP (pmol/L)	ROC (95% CI)	Sensitivity	Specificity
El-Khuffash et al. [29]	28	1.06	2	35	740	45	6046	4000	0.88 (0.79–0.96)	70%	89%
Nuntnarumit et al. [36]	30	1.30	2	23	463	12	1934	1203	0.96 (0.91–1.02)	100%	91%
Farombi et al. [37]	30	1.22	3	31	372	18	3891	1347	0.98 (0.93–1.03)	100%	95%
Ramakrishnan et al. [38]	29	1.22	2	36	1206	20	6952	2850	0.90 (0.81–0.99)	90%	89%
